# Evidence-based practice attitude scale for Latinx mental health professionals: a novel application of confirmatory factor analysis

**DOI:** 10.1186/s43058-025-00846-2

**Published:** 2026-03-09

**Authors:** Natalia Giraldo-Santiago, Julian M. Hernández-Torres, Daniel McNeish, Robin E. Gearing, Gregory A. Aarons

**Affiliations:** 1 Center for Health Outcomes and Interdisciplinary Research (CHOIR), Department of Psychiatry, Massachusetts General Hospital, Boston, MA USA; 2 Department of Psychiatry, Harvard Medical School, Boston, MA USA; 3 Medical Sciences Campus, Behavioral Sciences Research Institute, University of Puerto Rico, San Juan, Puerto Rico; 4 Department of Psychology, Arizona State University, Tempe, AZ USA; 5 Graduate College of Social Work, University of Houston, Houston, TX USA; 6 Department of Psychiatry, University of California, San Diego, CA USA; 7 Department of Psychiatry, Massachusetts General Hospital, One Bowdoin Square, Suite 100, Boston, MA 02114 USA

**Keywords:** Implementation Science, Evidence-Based Practice Attitudes Scale, Latinxs, Mental health professionals, Confirmatory factor analysis, Dynamic fit index cutoffs

## Abstract

**Background:**

The Evidence-Based Practice Attitude Scale (EBPAS) is a widely used measurement tool to assess mental health providers’ attitudes toward adopting research-based interventions. To date, this scale has not been used or validated in an interdisciplinary sample of mental health professionals in Latin America. This study investigated the factor structure, psychometric properties, cross-cultural validity, and model fit of the EBPAS in a sample of Spanish-speaking and Latino social workers, counselors, and psychologists.

**Methods:**

A culturally and linguistically tailored version of the 15-item EBPAS scale was administered to a sample of Puerto Rican mental health professionals (N = 222) working across various settings, including schools, healthcare clinics, and community organizations. The EBPAS’s scores were derived from four distinct constructs involving willingness to adopt EBPs (i.e., requirements, openness to innovation, appeal, and divergence from research). A Confirmatory Factor Analysis (CFA) examined the psychometric properties of the EBPAS scale. Several first and second-order factor models were specified. A global and approximate fit examination of the measurement model and composite reliability estimation for each subscale was conducted. RStudio version 4.3.1 software was used for the CFA.

**Results:**

The CFA supported a first-order factor model. Most subscales showed strong reliability coefficients ranging from 0.83 to 0.91, except for the divergence subscale, which showed a coefficient of 0.77**.** After allowing for covariance between two items in the appeal dimension, the correlated factor model demonstrated a satisfactory fit to the data, although some misspecification was observed.

**Conclusions:**

The tailored EBPAS-15 demonstrated adequate psychometric properties in this Latinx sample of mental health professionals, suggesting that its factor structure and reliability may be useful in a Spanish-speaking and Caribbean sample of mental health professionals working across a variety of settings and contexts. Findings contribute to the scant literature on culturally and linguistically validated measures examining attitudes toward EBPs in Latin America.

**Supplementary Information:**

The online version contains supplementary material available at 10.1186/s43058-025-00846-2.

Contributions to the literature

- Although there is a growing need for evidence-based practices (EBPs) among Latinx populations, there is a lack of culturally and linguistically tailored measures to examine attitudes towards their adoption and implementation.
- This is the first study to validate the Evidence-Based Attitudes Scale (EBPAS) with a sample of Latinx mental health professionals, representing a significant contribution to the field of implementation in Latin America.
- The application of Dynamic Fit Index (DFI) cutoffs for validating the psychometric properties of the Evidence-Based Attitudes Scale (EBPAS) represents a novel and innovative approach to latent variable modeling.

## Background

Evidence-Based Practices (EBPs) aimed at strengthening mental health services are becoming increasingly accessible, demonstrating their capacity to improve care quality and effectiveness across diverse populations [1, 2]. Yet, their implementation remains challenging in Latin America and Spanish-speaking countries [3]. Despite the growing prevalence of mental health disorders in the Latinx population, the adoption of EBPs among mental health professionals serving this vulnerable group remains significantly limited [4, 5]. A major barrier is the scarcity of culturally and linguistically adapted interventions with strong empirical support for their efficacy and real-world effectiveness among Latinx and Spanish-speaking individuals [6]. In the absence of translational and implementation efforts, some Latinx providers have expressed skepticism and resistance toward EBPs [7], along with concerns of services being “less individualized and more automated” [8].

The lack of culturally and linguistically tailored measures to assess providers’ attitudes toward delivering and implementing EBPs may hinder efforts to build the infrastructure and culture needed to advance scientific innovation in Latin American countries [6]. This challenge is especially pronounced in Puerto Rico, where mental health policies and professional organizations have relied on the use of traditional and pastoral approaches to mental health and addiction treatment [9]. However, research over the past decade shows a meaningful shift: mental health professionals are increasingly expressing a desire to move toward the use of evidence-based and culturally tailored treatments [4, 5, 10]. This trend highlights a critical opportunity to close implementation gaps by developing and validating measures that accurately capture mental health professionals’ attitudes toward EBP adoption in the region.

The Evidence-Based Practices Attitude Scale (EBPAS) is one of the most commonly used and psychometrically supported measures for assessing providers' attitudes towards EBPs [11]. However, this scale has yet to be validated among mental health professionals in Latin America. The EBPAS was developed as an initial effort to examine mental health providers’ attitudes toward adopting evidence-based practices (EBPs) in community mental health settings. Its development was informed by theoretical models suggesting that both individual attitudes and organizational factors (i.e., norms, expectations, and policies) serve as important precursors to decisions about whether providers will try, adopt, or sustain new practices [11].

The EBPAS scale has shown high reliability within the mental health field [11–13], the child welfare system [14], and school-based behavioral health settings [15]. Internationally, several studies have supported the EBPAS factor structure, reliability, and validity, notably in Norway [16, 17], Sweden [18], the Netherlands [19], Brazil [20], Germany [21], and Turkey [22]. In the United States, the EBPAS has been extensively examined across multiple service settings, consistently demonstrating strong reliability and validity among mental health professionals. Across these studies, the EBPAS has shown stable performance; however, most studies examining the psychometric properties of the EBPAS have been conducted with White and female samples in the US [12, 23, 24], underscoring the need for culturally and linguistically diverse validation efforts—particularly among Spanish-speaking, Latinx, and minority-serving provider groups.

Only two studies have adapted the EBPAS from English into Spanish. One translation was conducted with a European sample of social workers, psychologists, and educators working in the child welfare system in Spain [14], and the other with a diverse group of Latinx American clinical psychologists across 13 countries [25]. The latter provided preliminary evidence for a Spanish version in Latin American contexts, reporting acceptable to excellent internal consistency across the four EBPAS subscales. However, their study focused solely on clinical psychologists, excluded Puerto Rico, and did not examine validity, factor structure, or model fit. It was also unclear whether a standardized back-translation process was used. Importantly, no study to date has validated the EBPAS with an interdisciplinary sample in Puerto Rico, limiting the generalizability of existing findings.

This study evaluated the psychometric properties and suitability of the EBPAS among Latinx and Spanish-speaking mental health professionals in Puerto Rico. To do so, we adapted an existing Spanish version of the EBPAS that had previously undergone rigorous back-translation and preliminary psychometric testing in Castilian Spanish [14]. The EBPAS is well-suited for this study because it captures multiple dimensions of provider attitudes that are highly relevant to interdisciplinary mental health settings. This scale was selected based on evidence suggesting that individual and organizational factors (e.g., variation in training, professional roles, and socio-demographic characteristics) play a significant role in shaping mental health professionals’ attitudes toward adopting EBPs [4, 26].

## Methods

### Procedure and sampling

This study was approved by the University of Houston Institutional Review Board and recruited a convenience sample of licensed social workers, psychologists, and professional counselors in Puerto Rico. The principal investigator presented the study to leaders of three professional associations—the Professional Counselors Association (CPCR), the Psychology Association of Puerto Rico (APPR), and the College of Social Work Professionals (CPTSPR)—each of whom agreed to disseminate the study through their member listservs. The study flyer was also posted in Facebook groups for Puerto Rican mental health professionals. Due to initially low online response rates, additional in-person recruitment was conducted at a social work conference, where participants could complete the survey by scanning a QR code or using a printed version available on clipboards. Eligible participants were practicing mental health professionals residing in Puerto Rico and working in school, clinical, or community-based settings (e.g., child welfare). Participants received a five-dollar gift card as compensation.

### Participants

After screening, the final sample (N = 222) was predominantly female (83.1%) and Puerto Rican (96.8%), with a mean age of 44.34 years (SD = 10.30). Most participants were married (46.1%), held a master’s degree (65.3%), and had graduated from a private institution (73.1%). Annual salaries were generally low, with 42.4% earning under $30,000 and 25.6% earning $31,000–$40,000. Nearly one-third (29.2%) reported holding two jobs, 7.8% reported three jobs, and 5% reported four or more. The sample included social workers (47.7%), psychologists (28.4%), and professional counselors (23.9%). Over half worked in government settings (55.9%), while the remainder worked in private or mixed settings (44.1%), including nonprofit agencies and private practice. Practice settings varied and included schools (44.6%), clinical settings (22.0%), and community-based programs (12.4%). Participants were geographically distributed across metropolitan (48.4%) and non-metropolitan (51.6%) regions. Additional sociodemographic characteristics are presented in Table 1.

**Table 1:** Characteristics of the sample

Characteristics	Values (Percentages)
Age	
Mean (SD)	44.34 (10.30)
Gender	
Female	180 (82.2)
Male	39 (17.8)
Highest education	
Master’s degree	143 (65.3)
Doctorate degree	52 (23.7)
Something else	24 (11.0)
Income	
Less than 30 K	93 (42.4)
$31,000–40,000	56 (25.6)
$41,000–50,000	30 (13.7)
$51,000–60,000	17 (7.8)
More than 61 K	15 (6.8)
Prefer not to answer	8 (3.7)
Number of jobs	
One job	127 (58.0)
Two jobs	64 (29.2)
Three jobs	17 (7.8)
Four jobs or more	11 (5.0)

### Data handling

A total of 260 survey entries were collected. Eighteen entries were removed for completing the survey in under three minutes, and one entry was excluded after the respondent reported paying attention only “moderately” on an attention-check item. An additional 16 entries were removed due to implausible age–education discrepancies (e.g., reporting age 19 with a doctoral degree), which were likely generated by online bots, given that these responses originated from social media recruitment. Finally, three entries were excluded because no EBPAS items—the primary outcome—were completed. In total, 38 survey responses (approximately 1% of those who accessed the survey) were removed before analysis.

### Missing data

Additional measures were taken to prevent missingness. For example, each question required an answer before moving on. This feature reduced missing data but was not feasible for paper-based surveys. After screening, missing data were found for age (4.1%); marital status, gender, education, institution where education was acquired, number of jobs, income, ethnicity, and job location (1.4% each); practice setting (16.2%), and being of Latino (a) origin (18.0%). No other variables had missing data.

### Measure

The 15-item EBPAS has demonstrated strong psychometric properties in measuring both positive and ambivalent attitudes toward EBPs [11, 21, 23]. In 2017, the EBPAS scale was expanded to 36 items and validated in English and Norwegian [16], and subsequently validated in German [21]. More recently, a Spanish-European version was back-translated, adapted, and validated among child welfare professionals, demonstrating adequate reliability and validity [14]. In this study, the linguistic changes were minor and focused primarily on improving clarity and cultural relevance. Specifically, we tailored the Castilian Spanish EBPAS by consulting stakeholders familiar with the Puerto Rican context, linguistic nuances, and the study population. Based on their feedback, items 9–15 were revised to remove the clause “*if you received training in a therapy or intervention that was new to you*,” as stakeholders noted that this conditional phrasing implied access to training opportunities that may not exist in resource-constrained settings. The revised items more directly assessed attitudes toward adopting new therapies (e.g., “*I would adopt a new therapy or intervention if*…), thereby maintaining the original intent of an attitudes scale. Although most content from the validated Castilian version was retained, these minor adjustments were perceived to improve socio-cultural responsiveness without altering the underlying constructs. All modifications were cross-checked against the original English version to ensure conceptual and semantic equivalence. Table 2 provides a detailed comparison of the items in the original English, Castilian Spanish, and the tailored version used in this study.

**Table 2:** Comparative table across EBPAS items in the original English, Castilian Spanish, and the tailored version used in this study

Items	Original version in English (Aarons et al. 2004) [11]	Castilian Spanish Version (De Paul et al., 2015) [14]	Puerto Rican Spanish version (This study)
1	I like to use new types of therapy/interventions to help my clients	Me gusta utilizar nuevos tipos de intervenciones/terapias para ayudar a mis clientes	Me gusta utilizar nuevos tipos de intervenciones/terapias para ayudar a mis clientes
2	I am willing to try new types of therapy/interventions even if I have to follow a treatment manual	Estoy dispuesto/a a probar nuevas intervenciones/terapias……incluso si tengo que seguir un manual de tratamiento	Estoy dispuesto(a) a probar nuevos tipos de terapia/intervenciones aunque tenga que seguir un manual de tratamiento
3	I know better than academic researchers how to care for my clients	Yo sé cómo cuidar de mis clientes mejor que los investigadores	Yo sé cómo cuidar de mis clientes mejor que los investigadores
4	I am willing to use new and different types of therapy/interventions developed by researchers	Estoy dispuesto/a a probar nuevas intervenciones/terapias…… desarrollados por investigadores/as	Estoy dispuesto(a) a probar nuevas intervenciones/terapias desarrolladas por investigadores/as de salud mental
5	Research-based treatments/interventions are not clinically useful	Las intervenciones/tratamientos basados en la investigación no son útiles en la práctica clínica	Las intervenciones/tratamientos basados en evidencia no son útiles en la práctica clínica
6	Clinical experience is more important than using manualized therapy/interventions	La experiencia clínica es más importante que el utilizar intervenciones/terapias “guiadas por manual”	La experiencia clínica es más importante que el utilizar intervenciones/terapias guiadas por manual
7	I would not use manualized therapy/interventions	Yo no utilizaría intervenciones/terapias “guiadas por manual”	Yo no utilizaría intervenciones/terapias guiadas por un manual
8	I would try a new therapy/intervention even if it were very different from what I am used to doing	Estoy dispuesto/a a probar nuevas intervenciones/terapias incluso si fueran muy diferentes de lo que estoy acostumbrado a hacer	Estoy dispuesto (a) a probar nuevas intervenciones/terapias incluso si fueran muy diferentes a lo que estoy acostumbrado(a) a hacer
9	If you received training in a therapy or intervention that was new to you, how likely would you be to adopt it if:	Si recibiera formación en una terapia o intervención nueva para Vd., ¿con qué probabilidad la adoptaría si… fuera atractiva a primera vista?	Yo adoptaría una nueva terapia o intervención si esta fuera atractiva a primera vista
10	If you received training in a therapy or intervention that was new to you, how likely would you be to adopt it if: it was intuitively appealing?	“Si recibiera formación en una terapia o intervención nueva para Vd., ¿con qué probabilidad la adoptaría si… “tuviera sentido” para Vd.?	Yo adoptaría una nueva terapia o intervención si fuera fácil de entender
11	If you received training in a therapy or intervention that was new to you, how likely would you be to adopt it if: it was required by your supervisor?	“Si recibiera formación en una terapia o intervención nueva para Vd., ¿con qué probabilidad la adoptaría si… fuera requerido por su supervisor?	Yo adoptaría una nueva terapia o intervención si fuera requerida por mi supervisor (a)
12	If you received training in a therapy or intervention that was new to you, how likely would you be to adopt it if: it was required by your agency?	“Si recibiera formación en una terapia o intervención nueva para Vd., ¿con qué probabilidad la adoptaría si… fuera requerido por la entidad para la que trabaja?	Yo adoptaría una nueva terapia o intervención si fuera requerida por la agencia donde trabajo
13	If you received training in a therapy or intervention that was new to you, how likely would you be to adopt it if: it was required by your state?	Si recibiera formación en una terapia o intervención nueva para Vd., ¿con qué probabilidad la adoptaría si…fuera requerido por las autoridades de su comunidad autónoma?	Yo adoptaría una nueva terapia o intervención si fuera requerida a nivel isla
14	If you received training in a therapy or intervention that was new to you, how likely would you be to adopt it if: it was being used by colleagues who were happy with it?	Si recibiera formación en una terapia o intervención nueva para Vd., ¿con qué probabilidad la adoptaría …estuviese siendo utilizada por colegas que estuvieran contentos con su aplicación?	Yo adoptaría una nueva terapia o intervención si fuera utilizada por colegas contentos con su aplicación
15	If you received training in a therapy or intervention that was new to you, how likely would you be to adopt it if: you felt you had enough training to use it correctly?	Si recibiera formación en una terapia o intervención nueva para Vd., ¿con qué probabilidad la adoptaría si……Vd. sintiera que tiene la formación suficiente como para llevarla a cabo correctamente?	Yo adoptaría una nueva terapia o intervención si tuviera el adiestramiento suficiente como para llevarla a cabo correctamente

The present study administered the EBPAS-15 due to its brevity, practicality, and pragmatic characteristics [27]. The original 5-point Likert scale (0 = “not at all” to 4 = “to a very great extent”) was retained to maintain consistency with the validated response format. The EBPAS-15 comprises four first-order factors and one second-order (total) factor. The *appeal* subscale assesses the extent to which providers would adopt an EBP if it felt intuitively appealing, could be used correctly, or was endorsed by satisfied colleagues. The *requirements* subscale measures willingness to adopt EBP when mandated by an agency, a supervisor, or a broader system-level policy. The *openness* subscale evaluates general receptivity to new practices, including structured or manualized interventions. The *divergence* subscale captures the perception that EBPs are less useful or less important than clinical experience; items are negatively worded (e.g., “I know better than academic researchers how to care for my clients,” “research-based interventions are not clinically useful”). Higher divergence scores reflect less favorable attitudes toward EBPs. The second-order factor represents a global attitude toward EBPs, conceptualized as an overarching construct composed of the four subdimensions.

### Analyses

Statistical analyses were conducted in RStudio version 4.3.1 [28]. The scale was tested for multivariate normality using a test of multivariate normality implemented within the *MVN* package in R [29]. Visual examination of boxplots for each scale item was also considered to determine multivariate normality. These tests aided in deciding whether items should be modeled as continuous or categorical indicators [30, 31]. Summary statistics, item statistics, and Cronbach’s alpha [32] reliability coefficients for each subscale and the total scale were calculated using the *psych* package [33]. McDonald’s omega reliability coefficient [34, 35] was estimated for each subscale using the *MBESS* R package [36]. Bootstrap confidence intervals (CI) were computed for each coefficient and reported using a logit transform, as omega exists in a [0, 1] interval [37, 38]. For more details on the polychoric correlation matrix and standardized correlation residual matrix for the final model [see Additional files 1 and 2].

The use of Cronbach’s alpha for assessing scale reliability has been strongly dissuaded in the literature as it makes assumptions such as tau-equivalence (equal loadings) and uncorrelated item residuals [39–41]. These conditions are rarely met in practice, and thus the alpha coefficient tends to underestimate scale reliability to the extent that these assumptions are unmet. In these scenarios, Omega is more accurate than traditionally used methods (e.g., Cronbach's alpha) [42], and is thus reported alongside the alpha coefficient. Lastly, confirmatory factor analysis was conducted using the *lavaan* package [43] to examine the nature of relationships among latent constructs (e.g., requirement, openness to innovation, appeal, and divergence from research). The factor structures reported in the literature, as well as higher-order structures (hierarchical and bifactor models), were specified [11].

### Measurement model

A CFA [44] was performed to test the evidence-based factor structure proposed in Aarons [11]. Several additional models were specified and compared: (a) a correlated four-factor model, (b) a hierarchical factor model, (c) a bifactor model, and (d) a correlated factors model with correlated residuals. In model D, specifically the residuals for items nine, "I would adopt a new therapy or intervention if it was intuitively appealing," and ten, "I would adopt a new therapy or intervention as long as it made sense to me,*"* were correlated. Model D was evaluated, as it has been shown in the literature that items nine and 10 (Appeal subscale) tend to correlate due to similar wording [45].

A global and approximate fit examination of the measurement model was performed. To determine the exact global fit, the chi-square test of model fit was examined. To determine the approximate fit, the RMSEA, CFI, and SRMR were analyzed and compared across models. We chose to report only the CFI because both the CFI and the Tucker-Lewis Index (TLI) provide similar information, as they both measure incremental fit and are usually highly correlated [46]. Reporting both fit indices does not add any additional information. The TLI has also been known to produce fit index values greater than 1, which can be very difficult to interpret. On the other hand, the CFI is bounded between 0 and 1 which provides clearer indication of approximate fit [47].

Cutoffs for determining adequate model fit were derived using Dynamic Fit Index (DFI) cutoffs for CFA models [48]. In traditional CFA, good model fit is usually determined using cutoffs specified in the literature [49]. The performance of the different cutoff values can vary greatly based on aspects like sample size, factor reliability, number of items, and other model characteristics [40, 50, 51]. DFI cutoffs replicate the [50] method of constructing fit index cutoffs, but instead integrate user-specific model characteristics to create fit index cutoffs tailored to a particular model [49]. This method has been shown in simulation studies to produce cutoffs that correctly reject misspecified models and minimize the rejection of correctly specified models [48, 51, 52]. For a deeper explanation of how DFI cutoffs work and how they are interpreted, please refer to [52]. These cutoffs were derived using the *dynamic* package in R.

## Results

### Item statistics

A test of multivariate normality determined that the scale did not follow a multivariate normal distribution (*HZ* = 1.13, *p* < 0.001). Visual examination of boxplots revealed negative skewness for all items except three, six, and nine. Means and standard deviations for each item and estimates of subscale internal consistency (coefficient alphas and omegas) along with 95% CIs, item-total correlations for each subscale, and the total scale are reported in Table 3. Alpha reliability estimates ranged from 0.65 to 0.92. On the other hand, omega reliability estimates ranged from 0.69 to 0.93.

**Table 3:** Summary statistics of subscale, item means, and standard deviations

				*Sub-scale*	*Total scale*
EBPAS subscales	M (SD)	α [95% CI]	ω [95% CI]	*r*	*r*
**Requirement**		.92 [.90,.93]	.93 [.89,.96]		
Agency required EBPAS 12	2.46 (1.09)			.94	.64
Supervisor required EBPAS 11	2.22 (1.14)			.93	.62
Island-wide required EBPAS 13	2.45 (1.08)			.91	.67
**Appeal**		.77 [.71,.81]	.81 [.76,.86]		
Make sense EBPAS 10	2.61 (1.04)			.83	.60
Intuitively appealing EBPAS 9	2.14 (1.18)			.81	.54
Colleagues happy with therapy EBPAS 14	2.46 (1.12)			.81	.56
Enough training to use it correctly EBPAS 15	3.41 (0.82)			.60	.54
**Openness**		.85 [.81,.88]	.86 [.82,.89]		
Will follow a treatment manual EBPAS 2	3.09 (0.93)			.86	.61
Therapy developed by researchers EBPAS 4	3.14 (0.87)			.86	.61
Like new therapy types EBPAS1	2.95 (1.06)			.82	.54
Therapy different than usual EBPAS 8	2.88 (1.08)			.79	.65
**Divergence**		.65 [.57,.72]	.69 [.60,.76]		
Research-based treatments not useful EBPAS 5	3.08 (1.27)			.75	.31
Would not use manualized therapy EBPAS 7	2.84 (1.16)			.70	.24
Clinical experience more important than manualized interventions EBPAS 6	2.16 (1.11)			.68	.25
Know better than researchers EBPAS 3	2.18 (1.18)			.67	.09
**EBPAS Total**	40.07 (7.88)	.77 [.72,.81]	.89 [.64,.97]		

Cronbach’s alpha, McDonald’s omega, and item-total correlations reported; see Additional File 1 for Spanish items

### Model fit

Diagonally Weighted Least Squares was used for parameter estimation, as it accounts for the categorical nature of Likert-type items [45]. The information matrix could not be inverted in Models B and C, so parameter estimates were unavailable because the optimization algorithm did not converge. Models B and C were not further evaluated. However, optimization converged without issue for all correlated factor models. Fit statistics for model A were χ^2^ (df = 84) = 238.34, *p* < 0.001, CFI = 0.991, RMSEA = 0.091 (90% CI: [0.078, 0.105]), and SRMR = 0.084. Fit statistics for model D were χ^2^ (*df* = 83) = 205.08, *p* < 0.001, CFI = 0.993, RMSEA = 0.082 (90% CI: [0.068, 0.096]), and SRMR = 0.081.

None of the models satisfied the Level-0 DFI cutoff, and significant χ2 tests indicate that neither model demonstrated exact fit. For Model A, CFI satisfied the Level-3 DFI cutoff, RMSEA satisfied the Level-3 DFI cutoff, and SRMR satisfied the Level-2 DFI cutoff. For Model D, CFI satisfied the Level-3 DFI cutoff, RMSEA satisfied the Level-3 DFI cutoff, and SRMR satisfied the Level-1 DFI cutoff. Collectively, this indicates that Model A and Model D generally demonstrate reasonable approximate fits, given the specific model and data characteristics. Cutoffs derived for Models A and D are presented in Table 4.

**Table 4:** Dynamic fit index cutoffs per level of misspecification

	**Interpretation**	**SRMR**	**RMSEA**	**CFI**
**Model A**				
Level-0	Fit index values if fitted model were the underlying population model	0.055	0.000	1.000
Level-1	Fit index values if model contains a misspecification consistent with an omitted cross-loading of 0.562 in magnitude	0.081	0.064	0.993
Level-2	Fit index values if model contains cumulative misspecification consistent with omitted cross-loadings of 0.562 and 0.385 in magnitude	0.085	0.072	0.993
Level-3	Fit index values if model contains misspecification consistent with omitted cross-loadings of 0.562, 0.385, and 0.423 in magnitude	0.095	0.096	0.989
**Model D**				
Level-0	Fit index values if fitted model was the true population model	0.056	0.000	1.000
Level-1	Fit index values if model contains a misspecification consistent with an omitted cross-loading of 0.562 in magnitude	0.082	0.065	0.993
Level-2	Fit index values if model contains cumulative misspecification consistent with omitted cross-loadings of 0.562 and 0.370 in magnitude	0.084	0.070	0.994
Level-3	Fit index values if model contains cumulative misspecification consistent with omitted cross-loadings of 0.562, 0.370, and 0.424 in magnitude	0.094	0.095	0.990

Specifically, based on both CFI and RMSEA, both models appear to have cumulative misspecification below three omitted cross-loadings, with magnitudes in the mid-0.50 s, high-0.30 s, and mid-0.40 s. Based on SRMR, model A had cumulative misspecification consistent with a single omitted cross-loading of magnitude within the mid-0.50 s. On the other hand, model D had cumulative misspecification below a single omitted cross-loading whose magnitude was in the mid-0.50 s. Both models satisfy the same DFI cutoff levels, with model D showing less misspecification according to the SRMR. In addition, Model D’s factor structure appeared to explain the inter-item correlations slightly better than Model A.

### Factor structure

In addition to global fit, local fit was also evaluated by inspecting each element of the standardized residual matrix [53, 54]. In general, elements of the standardized residual matrix with absolute values below 0.10 indicate that the model closely reproduces a particular observed correlation [55]. Clusters of larger values can indicate potential areas of local strain in the model and help identify potential specifications [56]. In Model A and Model D, 27% and 25% of the standardized residual matrix elements, respectively, exceed an absolute value of 0.10. There was some limited evidence of local strain involving Item 15 (Appeal) in both models (specifically, the correlation between Items 7–9 and Item 15). However, like the global fit assessment, the local fit assessment also suggests a reasonable fit of the model.

**Fig. 1:**
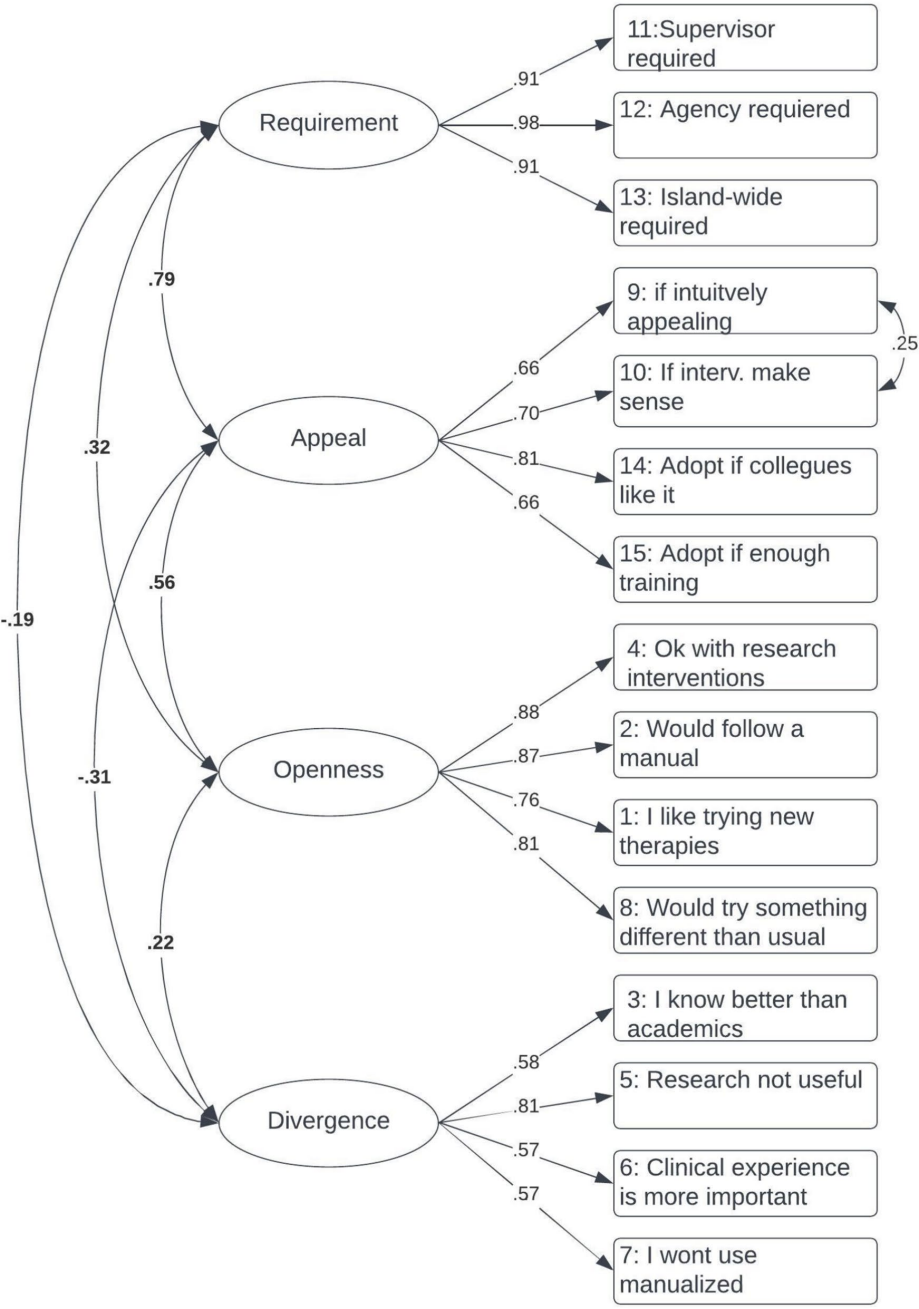
Confirmatory Factor Analysis results for model D. Note. Fully standardized estimates are shown

Figure 1 presents the factor loadings, residual correlations, and factor correlations for model 4. Factor correlations ranged from −0.19 to 0.79. Factor loadings were moderate-to-strong and positive, ranging from 0.57 to 0.98. Appeal had a moderately positive correlation with Openness and a significantly negative correlation with Divergence. A highly positive correlation was observed between the Appeal and Requirement scales. However, a weak negative correlation between Divergence and Requirement and a weak positive correlation between Divergence and Openness were observed.

## Discussion

Our study addressed a critical gap by validating the EBPAS in an interdisciplinary sample of social workers, counselors, and psychologists across school, clinical, and community settings in Puerto Rico. The EBPAS-15 demonstrated adequate psychometric properties in this Spanish-speaking Caribbean sample, supporting its reliability, validity, and applicability. Notably, the factor structure, which aligns with Aarons’ original conceptual model, positing that attitudes toward EBPs are multidimensional and influenced by both individual and organizational factors, is highly relevant to Puerto Rico’s resource-constrained, community-based mental health workforce. This is the first study to provide robust psychometric evidence for a linguistically tailored EBPAS in Puerto Rico and among an interdisciplinary sample of Latinx mental health professionals.

Findings were consistent with prior CFA studies conducted on the EBPAS scale [18, 25]. Constructs involving willingness to adopt EBPs, given their intuitive appeal, inclination to adopt new practices if required, and general openness toward new or innovative practices, performed well. The subscales referring to appeal, openness, and requirement had strong reliabilities of 0.81, 0.86, and 0.93, respectively. These measures performed well, as evidenced by the strong positive correlation between the Appeal dimension and Requirement and the moderate positive correlation with Openness. However, the divergence subscale did not perform as expected. The factor covariances between the Divergence and Appeal subscales differed from those reported in the original CFA [11]. For example, a negative rather than a positive correlation between the Divergence and Appeal subscales was observed [20]. Providers with greater divergence from research were more likely to adopt EBPs based on how appealing the intervention was to them and their colleagues. Interestingly, divergence from research did not result in negative attitudes toward adopting an intervention if the intervention made sense to them or if they had sufficient training. It is possible that the appeal of an intervention may have more meaning and direct influence on their attitudes than the evidence reported from research studies. This could be because mental health professionals interact with research much less than patients, which could increase attitudes of divergence.

As in prior studies, a significant but weak negative correlation was found between the Divergence and Requirement subscales [25]. The weak negative correlation suggests that as the perceived requirement to adopt EBPs increases, the perceived divergence or flexibility of those practices tends to decrease, or vice versa. However, the small effect of this correlation highlights the importance of carefully identifying and working with resistance to federal laws requiring the adoption of EBPs in Puerto Rico, and examining how socio-economic, educational, and cultural factors influence the relationship between divergence and requirement [4]. This is important, as previous studies have found that income plays a role in attitudes towards research divergence, and access to educational and training gaps has been found to correlate with greater use of interventions with well-established, probably efficacious evidence [26].

Contrary to the literature, a non-zero positive correlation was found between the Divergence and Openness subscales. This correlation reached a magnitude of 0.22, compared to Aaron’s finding of 0.03 [11]. Although the relationship between these constructs remained weak, the directionality of the covariance suggests that participants with greater divergence from research were also more likely to be open to innovation, which is counterintuitive. The limited research on EBPs raises valid concerns that mental health professionals may diverge from practices often developed and adapted by non-Latinx individuals in the mainland US [26]. However, their continued openness to innovation offers some grounds for optimism. It underscores the need for a more nuanced examination of attitudes toward EBPs among mental health professionals in Puerto Rico. It is possible that the EBPAS scale may not fully capture the socio-cultural and political factors driving divergent attitudes towards research. Thus, we strongly recommend using additional measures (e.g., the expanded EBPAS 36-item scale) and methods to assess implementation attitudes, as well as barriers and facilitators.

Our study findings are different from the EBPAS Spanish version used in Toro and Montoya (2019), which only had reliability above 0.80 in the Requirement subscale [25]. Their study reported higher reliability estimates for the Divergence subscale and lower reliability for the Appeal subscale, compared with our results. We believe our adapted version of the EBPAS scale and thus our results are much closer to those seen in Aarons (2004) and De Paul et al. (2015) [11, 14]. Both studies showed the Divergence scale had less than acceptable reliability, excellent reliability for the Requirement subscale, and overall evidence of good fit. It is worthy of note that the version of the EBPAS in Toro and Montoya (2019) does not have the same item structure as in Aarons' (2004) original version, and the version developed for this study [11, 25].

The low to adequate reliability in the divergence from research subscale is consistent with studies conducted in Norway, Brazil, the Netherlands, and Germany, indicating a weaker reliability of the Divergence subscale [16, 17, 19–21]. In this Puerto Rican sample, the inconsistent reliability findings in the divergence from the research dimension might be related to different terms utilized in the scale. For example, differences were noted in the size of the loadings between the two items referring to "manualized therapy/interventions" and the two items using the term "research-based treatments/interventions." These findings suggest an opportunity to explore whether mental health professionals have any preferences in the terminology used to refer to EBPs, and whether the usage of manualized therapies might be more appealing than research-based interventions.

Overall, the CFA results indicated that the four-factor EBPAS model was appropriate. Higher-order factor structures were supported in some countries (e.g., Germany and the Netherlands), whereas other studies examined only first-order models. Differences in study design and methodologies across the literature warrant further cross-cultural validation to ensure the EBPAS functions consistently across this diverse professional sample in a new linguistic setting. Furthermore, second-order models were not empirically supported, putting into question the presence of a general, overarching *evidence-based practice attitude* dimension in this population. However, this does not imply that the utilization of a total score is of no practical use. Findings show empirical support for all subscales.

The adequacy of the EBPAS scale holds promising potential for advancing implementation science in Puerto Rico. However, these efforts should take into account the local context, as well as the individual and organizational characteristics and needs that could facilitate the adoption of EBPs in the region [4]. In fact, findings from this study should be considered in the context of Puerto Rico's status as a US territory in the Caribbean, which presents unique challenges for implementing EBPs. For instance, Puerto Rico’s socio-political and economic vulnerability has limited scientific advancements in the past decade and left many organizations without resources to improve the skillset of their mental health professionals [4]. This scarcity of cost-effective, accessible, evidence-based programs in Spanish, as well as the lack of educational and training resources, has left many mental health professionals with resistance to research, concerns about adopting practices that are not accessible, and with thoughts of migrating to the US mainland for more training opportunities on EBPs [57].

Based on this context and the findings of this study, we offer concrete recommendations to improve the training of mental health professionals and the dissemination of EBPs in Latin America. First, organizations must prioritize evaluating and implementing socio-cultural practices perceived to be working and/or showing preliminary evidence of probably efficacious treatments [6]. Second, there is a need to engage mental health professionals with existing resources and training to conduct adaptations of existing manualized and research-based interventions with probably efficacy in the Puerto Rican community. Third, terms such as "manualized therapies" or "research-based treatments" should be used carefully, as they may be perceived negatively, particularly if there is no context indicating whether these treatments have been tested and adapted for Puerto Rican populations. Fourth, research and clinical initiatives should explore which resources, training materials, and educational opportunities are attractive and perceived as beneficial for their development on EBPs.

To gain a deeper understanding of the challenges that may arise in the adoption of EBPs in Latin America, future research could employ explanatory concurrent and sequential mixed method designs to examine attitudes towards EBPs, using both quantitative and qualitative approaches. This type of research will be critical for understanding the context that influences the adoption of EBPs, as well as stakeholders’ perspectives on strategies to overcome challenges. Through in-depth interviews, researchers and organizations can thoughtfully consider the resources available and the needs to be addressed when implementing EBPs [6]. In sum, we believe that culturally and linguistically based methods to assess attitudes towards the adoption of EBPs are a critical step to move away from traditional, non-empirical, and sometimes harmful mental health practices implemented in the region.

### Limitations

Findings should be interpreted with caution, as Divergence from research had a reliability of 0.69, which was acceptable but not ideal. Consistent with a psychometric study of this measure in a large sample of Swedish mental health professionals [45], each subscale for Spanish-speaking and Latinx mental health providers demonstrated good internal consistency and reliability, except for one subscale that showed divergence. The Divergence subscale was not a substantial measure of resistance to engaging in EBPs among mental health professionals in Puerto Rico. A possible limitation of this subscale was the negatively worded items, such as *"*I would not use manualized therapy/interventions" and “Research-based treatments/interventions are not clinically useful.” Furthermore, some of these statements could be perceived as gauges or questions of the provider’s expertise and clinical experience. Future research could examine the role of clinical practice, expertise, and experiences in the adoption and implementation of EBPs.

Another limitation of this measure was the possible difficulty in responding to a Likert scale ranging from “not at all” to “a very great extent” when measuring attitudes. We suspect that this type of wording could have increased the chances of misunderstanding the direction of an item. A Likert scale focused on measuring levels of agreement (i.e., *strongly agree* to *strongly disagree*) could be linguistically and culturally more suitable, but this hypothesis should be empirically tested. It is recommended that future studies conduct cross-cultural back-translation of the English EBPAS measure into Latin American Spanish using bilingual individuals from the region and replicate this study in other Latinx and Spanish-speaking samples, such as US-based Latinxs. While this tailored measure could be used in Latin America, the language was tested only with Latinx-identifying individuals residing in Puerto Rico.

We encourage future studies to explore how the conceptual framework behind attitudes towards EBPs is understood in the Puerto Rican context, especially the Divergence construct. Specifically, future work could examine concordances or discrepancies between the expanded EBPAS-36 item and the original 15-item EBPAS. Lastly, future studies should employ multi-trait multi-method analyses to examine variance between methods, as we did not account for survey modality effects or participants’ occupations.

### Strengths

This study had several strengths in its statistical and methodological design. First, this study provides the first reliability and validity evidence for the EBPAS in Puerto Rico and Latin American/Caribbean contexts. It also studied diverse types of professional and educational backgrounds and workplace settings. Second, a back-translated EBPAS measure from English to Castilian Spanish was tested and modified before launching the survey. Minor changes to certain words were made to improve the readability among a Puerto Rican sample. Third, this study is innovative in that it gathers attitudes towards EBPs from a multidisciplinary, diverse sample. Additional strengths reside in the statistical approaches chosen to analyze the data. DFI cutoffs are a valuable alternative to traditional, rule-of-thumb cutoffs for fit evaluation in CFA [53, 54]. However, DFI cutoffs have seldom been used in implementation sciences research, a novel contribution to the field of implementation science psychometrics. Thus, further exploration of this method and its potential applications is warranted. Cutoffs used in this study reflect the intricacies of our model, thereby enabling a more accurate and in-depth assessment of model fit, informing a more in-depth approach to model adequacy.

## Conclusions

In this study, we adapted the European Spanish version of the EBPAS scale to Caribbean Spanish for a Latinx sample, yielding strong psychometric properties. This effort addresses a critical need for reliable, culturally validated measures to facilitate the implementation of Evidence-Based Practices (EBPs) in Latin America, particularly in Puerto Rico, where their adoption has been slow. The EBPAS measure offers important methodological guidance for researchers, program managers, and practitioners evaluating and interpreting attitudes toward the adoption of scientifically driven interventions, policies, and practices in Latin America. This process should be supported by psychometrically robust, cross-culturally validated measures, such as the Evidence-Based Practice Attitude Scale (EBPAS), alongside qualitative methods to yield reliable, contextually relevant insights.

## Supplementary Information

Additional file 1.

Additional file 2.

## Data Availability

The datasets analyzed in the current study are available in the Open Science Framework repository at osf.io/58ctv.
