# Supplementary material for: Evidence-based practice attitude scale for Latinx mental health professionals: a novel application of confirmatory factor analysis
Source: Implement Sci Commun. 2026 Mar 9;7:73. doi: 10.1186/s43058-025-00846-2 (PMC13081654; doi:10.1186/s43058-025-00846-2)
Supplement: Supplementary file 1 — Additional file 1. [file 43058_2025_846_MOESM1_ESM.docx]

**Additional file 1**

| *Polychoric correlation matrix* | | | | | | | | | | | | | | | |
| --- | --- | --- | --- | --- | --- | --- | --- | --- | --- | --- | --- | --- | --- | --- | --- |
|  | EBPAS1 | EBPAS2 | EBPAS3 | EBPAS4 | EBPAS5 | EBPAS6 | EBPAS7 | EBPAS8 | EBPAS9 | EBPAS10 | EBPAS11 | EBPAS12 | EBPAS13 | EBPAS14 | EBPAS15 |
| EBPAS1 | 1.00 |  |  |  |  |  |  |  |  |  |  |  |  |  |  |
| EBPAS2 | 0.75 | 1.00 |  |  |  |  |  |  |  |  |  |  |  |  |  |
| EBPAS3 | -0.03 | 0.00 | 1.00 |  |  |  |  |  |  |  |  |  |  |  |  |
| EBPAS4 | 0.66 | 0.78 | -0.03 | 1.00 |  |  |  |  |  |  |  |  |  |  |  |
| EBPAS5 | 0.29 | 0.28 | 0.39 | 0.21 | 1.00 |  |  |  |  |  |  |  |  |  |  |
| EBPAS6 | 0.14 | 0.20 | 0.35 | 0.02 | 0.46 | 1.00 |  |  |  |  |  |  |  |  |  |
| EBPAS7 | 0.09 | 0.15 | 0.32 | 0.06 | 0.51 | 0.33 | 1.00 |  |  |  |  |  |  |  |  |
| EBPAS8 | 0.54 | 0.61 | -0.03 | 0.70 | 0.21 | 0.07 | 0.02 | 1.00 |  |  |  |  |  |  |  |
| EBPAS9 | 0.23 | 0.24 | -0.32 | 0.30 | -0.12 | -0.19 | -0.10 | 0.37 | 1.00 |  |  |  |  |  |  |
| EBPAS10 | 0.26 | 0.30 | -0.25 | 0.33 | -0.01 | -0.09 | -0.05 | 0.44 | 0.71 | 1.00 |  |  |  |  |  |
| EBPAS11 | 0.10 | 0.20 | -0.20 | 0.23 | -0.15 | -0.09 | -0.07 | 0.35 | 0.49 | 0.50 | 1.00 |  |  |  |  |
| EBPAS12 | 0.12 | 0.21 | -0.14 | 0.27 | -0.13 | -0.06 | -0.11 | 0.35 | 0.46 | 0.50 | 0.87 | 1.00 |  |  |  |
| EBPAS13 | 0.21 | 0.20 | -0.14 | 0.29 | -0.01 | -0.09 | -0.13 | 0.35 | 0.54 | 0.55 | 0.81 | 0.83 | 1.00 |  |  |
| EBPAS14 | 0.16 | 0.16 | -0.24 | 0.27 | -0.24 | -0.18 | -0.16 | 0.35 | 0.57 | 0.59 | 0.63 | 0.64 | 0.59 | 1.00 |  |
| EBPAS15 | 0.33 | 0.44 | -0.20 | 0.50 | 0.02 | 0.04 | 0.07 | 0.49 | 0.25 | 0.43 | 0.40 | 0.42 | 0.47 | 0.54 | 1.00 |
