# Supplementary material for: Evidence-based practice attitude scale for Latinx mental health professionals: a novel application of confirmatory factor analysis
Source: Implement Sci Commun. 2026 Mar 9;7:73. doi: 10.1186/s43058-025-00846-2 (PMC13081654; doi:10.1186/s43058-025-00846-2)
Supplement: Supplementary file 2 — Additional file 2. [file 43058_2025_846_MOESM2_ESM.docx]

*Evidence-Based Practice Attitude Scale (EBPAS-15) Spanish Version adapted for this study*

Las siguientes preguntas buscan conocer su opinión sobre el uso de nuevos tipos de terapias, intervenciones o tratamientos manualizados de salud mental. Las terapias o tratamientos manualizados son intervenciones con directrices y componentes específicos que se recogen en un manual y que deben seguirse de forma estructurada o predeterminada. Indique en qué medida está de acuerdo con cada punto utilizando la escala provista.

1= Para nada

2= En un grado leve

3= En un grado moderado

4= En gran medida

5= Totalmente

1. Me gusta utilizar nuevos tipos de intervenciones/terapias para ayudar a mis clientes.

2. Estoy dispuesto(a) a probar nuevos tipos de terapia/intervenciones aunque tenga que seguir un manual de tratamiento.

3. Yo sé cómo cuidar de mis clientes mejor que los investigadores. (R)

4. Estoy dispuesto(a) a probar nuevas intervenciones/terapias desarrolladas por investigadores/as de salud mental.

5. Las intervenciones/tratamientos basados en evidencia no son útiles en la práctica clínica. (R)

6. La experiencia clínica es más importante que el utilizar intervenciones/terapias guiadas por manual.(R)

7. Yo no utilizaría intervenciones/terapias guiadas por un manual.

8. Estoy dispuesto (a) a probar nuevas intervenciones/terapias incluso si fueran muy diferentes a lo que estoy acostumbrado(a) a hacer.

9. Yo adoptaría una nueva terapia o intervención si esta fuera atractiva a primera vista.

10. Yo adoptaría una nueva terapia o intervención si es fácil de entender.

11. Yo adoptaría una nueva terapia o intervención si fuera requerida por mi supervisor (a).

12. Yo adoptaría una nueva terapia o intervención si fuera requerida por la agencia donde trabajo.

13. Yo adoptaría una nueva terapia o intervención si fuera requerida a nivel isla.

14. Yo adoptaría una nueva terapia o intervención si fuera utilizada por colegas contentos con su aplicación.

15. Yo adoptaría una nueva terapia o intervención si tuviera el adiestramiento suficiente como para llevarla a cabo correctamente.
